# Soluble P-Selectin as an Indicator of Cutaneous Microangiopathy in Uncomplicated Young Patients with Type 1 Diabetes

**DOI:** 10.3390/life14121587

**Published:** 2024-12-02

**Authors:** Jolanta Neubauer-Geryk, Małgorzata Myśliwiec, Katarzyna Zorena, Leszek Bieniaszewski

**Affiliations:** 1 Clinical Physiology Unit, Medical Simulation Centre, Medical University of Gdańsk, 80-210 Gdańsk, Poland; lbien@gumed.edu.pl; 2 Department of Pediatrics, Diabetology and Endocrinology, Medical University of Gdańsk, 80-211 Gdańsk, Poland; malgorzata.mysliwiec@gumed.edu.pl; 3 Department of Immunobiology and Environment Microbiology, Medical University of Gdańsk, 80-211 Gdańsk, Poland; kzorena@gumed.edu.pl

**Keywords:** adhesion molecules, sP-selectin, cutaneous microcirculation reactivity, pulsatility indices, ankle–brachial index, endothelium dysfunction, capillaroscopy, type 1 diabetes mellitus, children and adolescents

## Abstract

This study aimed to analyze the relationship between cutaneous microcirculation reactivity, retinal circulation, macrocirculation function, and specific adhesion molecules in young patients with uncomplicated type 1 diabetes. Fifty-five patients with type 1 diabetes mellitus (T1DM), aged 8 to 18 years, were divided into subgroups based on skin microcirculation reactivity. The cutaneous microcirculatory vessels were considered reactive if post-test PORH coverage increased compared to pre-test coverage. Optical coherence tomography (OCT) was conducted to detect early retinopathic changes. Macrocirculation was described using pulsatility indices (PIs) determined for common carotid (CCA) and peripheral arteries of the upper and lower limbs. The ankle–brachial index was also assessed. There were no significant differences in retinal circulation and macrocirculation between the studied subgroups. However, there were significant differences between the various subgroups concerning the age at onset of diabetes and the sP-selectin levels but not ICAM-1 and sVCAM-1. The sP-selectin differences remained true after adjusting for age at onset. The sP-selectin level was significantly higher in the subgroup of patients with non-reactive cutaneous microcirculation. The results of our study indicate that sP-selectin may be considered as an immunological marker for cutaneous abnormalities, which serve as an early indicator of endothelial dysfunction in young patients with type 1 diabetes in the absence of classical complication.

## 1. Introduction

Endothelial dysfunction is an established feature of type 1 diabetes (T1DM) [1] and is linked to a spectrum of classical microvascular complications (retinopathy, neuropathy, and nephropathy) [2] and non-classical complications (brain, lung, bone tissue, skin, arterial wall, heart, or musculoskeletal) [3]. Therefore, the presence of complications can signify an advanced stage of deterioration in capillary function and associated structural changes.

The literature indicates a correlation between the progression of microvascular alterations assessed using capillaroscopy and glycemic control in individuals with diabetes [4,5,6,7]. Some reports indicate a relationship between the observed abnormalities in capillary imaging and the clinical presentation of microangiopathy in diabetes. Kuryliszyn-Moskal et al. established a correlation between the degree of alterations observed in capillary morphology and metabolic control, as well as the presence of chronic complications, in patients diagnosed with T1DM [6,7]. However, in Gasser’s study [5], no significant difference was observed between subjects with healthy capillary function and those with T1DM when capillary blood flow was assessed using video capillary microscopy of the fingernail bed under resting conditions and after local cooling. Forst demonstrated that individuals diagnosed with T1DM and peripheral neuropathy did not exhibit significant changes in acetylcholine-stimulated blood flow [4].

Qualitative microvascular abnormalities observed using capillaroscopy are common (81%) among adult patients with type 1 diabetes [6]. In our previous report, we showed that T1DM has a markedly negative impact on microvascular reactivity, as evaluated using capillaroscopy and l-arginine stimulation [8] or the post-occlusion reactive hyperemia test (PORH) [9]. Cutaneous microcirculation serves as a pivotal and well-established model for examining the impact of various factors on global microcirculatory function [10].

From a young age, arterial walls undergo a progressive stiffening process due to a loss of elastic fibers. The presence of metabolic disorders, such as hyperlipidemia or hyperglycemia [11], accelerates this process. Arterial elasticity is associated with pulsatility, representing an intrinsic property and a fundamental aspect of the cardiovascular system. The elasticity of blood vessels permits the attenuation of damaging pulsations in major arteries prior to their propagation to the microcirculation of distal organs. Diminished elasticity is linked to unfavorable outcomes, including increased stress on the left ventricle, progressive elevation in blood pressure, and ultimately damage to end organs by transmitting harmful pulsations into the microcirculation. It was shown that in T1DM patients, increased pulsations resulting from large vessel stiffness affect capillary networks, thereby causing microvascular complications [12]. In light of this, several studies have demonstrated the feasibility of using the pulsatility index in the study of larger blood vessels, allowing for the prediction and prevention of microvascular complications in patients with diabetes. The pulsatility index (PI) of the internal carotid artery was significantly correlated with the occurrence of microalbuminuria in patients with type 2 diabetes mellitus (T2DM) [13]. Furthermore, PI may serve as an informative indicator for the prediction of renal dysfunction in individuals with T2DM. Lee et al. demonstrated that the PI in the middle cerebral artery and internal carotid artery was markedly elevated in patients with microvascular complications, including retinopathy, nephropathy, and neuropathy, compared to subjects without complications and healthy controls [14]. Moreover, the pulsatility index has been utilized to establish the impact of specific therapeutic interventions in individuals with T2DM [15].

The initial damage to the endothelium triggers a prompt endothelial activation process independent of protein synthesis and gene transcription. These processes result in hemorrhage and edema, which lead to the release of protective proteins such as von Willebrand factor (vWF), P-selectin, thrombin, and histamine. The subsequent phase of endothelial activation is prolonged and dependent on protein synthesis, ultimately resulting in the release of P-selectin, ICAM-1, VCAM-1, vWF, etc.

The proatherogenic effects of hyperglycemia have been identified as resulting from endothelial activation. The increased expression of cell adhesion molecules, including P-selectin, E-selectin, and VCAM-1 [16], has been observed to result in endothelial dysfunction, characterized by the apoptosis and necrosis of endothelial cells, which are considered to be hallmarks of endothelial damage [17]. Cell adhesion molecules, including selectins, have been demonstrated to serve as reliable biomarkers for microvascular complications, including retinopathy, nephropathy, and neuropathy, in individuals with T1DM [7] as well as T2DM [18,19]. The elevated levels of soluble P-selectin observed in patients with atherosclerosis risk factors are not as obvious as those observed in patients with overt disease. The results of studies conducted by numerous research groups have corroborated the presence of elevated levels of P-selectin in a multitude of acute and chronic cardiovascular conditions [20].

The present study aimed to study the relationship between adhesion molecule levels and cutaneous microcirculation reactivity as well as measures of circulatory dysfunction in other vascular regions, i.e., the retina, where OCT (optical coherence tomography) was utilized to identify any abnormalities, and large vessel dysfunction, where PI was employed to quantify the extent of impairment in T1DM. To identify the earliest evidence of microcirculation dysfunction, our study group was limited to young non-complicated T1DM patients. Our study investigates whether abnormalities of the cutaneous microcirculation may precede the onset of typical microangiopathies.

## 2. Materials and Methods

### 2.1. Study Group

The study population included 55 young patients with type 1 diabetes, aged 15.3 ± 2.4 years with a disease onset of 7.8 ± 3.5 years and a disease duration of 7.4 ± 3.7 years. The median BMI was 20.8 (14.5–29.7) kg/m^2^, the median HbA_1c_ level was 7.9 (5.9–13.4) %. The total percentage of male subjects within the group was 47%. All patients were treated at the Department of Pediatrics, Diabetology and Endocrinology, University Clinical Center in Gdansk, and met the diagnostic criteria for type 1 diabetes according to the criteria set forth by the International Society of Child and Adolescent Diabetes (ISCAD) [21].

The exclusion criteria included micro- and macroangiopathic complications, acute complications of diabetes, abnormal thyroid-stimulating hormone (TSH) and free thyroxine levels, and systemic diseases such as rheumatoid arthritis and psoriasis. Additionally, the use of statins was excluded. The exclusion of diabetic retinopathy was based on an ophthalmoscopic evaluation of the fundus after pupil dilation by an ophthalmologist, conducted following the criteria set forth by the American Diabetes Association [22]. A diagnosis of diabetic neuropathy was based on the coexistence of subjective and objective symptoms of neuropathy, as detailed in the corresponding literature [23]. The presence of diabetic nephropathy was determined based on the results of albuminuria tests conducted within the six months prior to the study, in addition to the current determination of albumin levels in the collected urine sample. Patients with diabetic ketoacidosis at the time of enrollment, ongoing infection, uncontrolled celiac disease, or chronic kidney disease were excluded from participation in the study. Additionally, patients who had experienced severe hypoglycemia within the previous month were excluded from the study. Severe hypoglycemia was defined as an episode of blood glucose concentration below 54 mg/dL that required the intervention of another individual. An episode of mild hypoglycemia was defined as a blood glucose level of less than 54 mg/dl that did not result in the need for intervention [24]. Following the presentation of the study’s objectives and methodology, the participants and their parents provided informed consent. The research methodology was approved by the Independent Bioethics Committee for Scientific Research at GUMed (decisions NKBBN/277/2014 dated 8 July 2014, and NKBBN/277-512/2016 dated 5 December 2016).

### 2.2. Laboratory Analysis

Blood samples for laboratory analysis were collected from subjects fasting between 7 and 9 a.m. The serum was separated from the venous blood and stored at −80 °C for three months before analysis.

All measurements were based on analysis of the same blood sample. HbA_1c_ levels were quantified through immunoturbidimetric measurement using the UniCel DxC 800 Synchron System (Beckman Coulter, Brea, CA, USA) with the Uni-H1A3 assay kit (Roche Diagnostics, Basel, Switzerland). Fasting glucose levels were assessed via an enzyme-based assay (Roche Diagnostics GmbH, Mannheim, Germany).

The concentration of C-reactive proteins was determined by an immunochemical assay utilizing the Beckman Instruments, Inc. (Galway, Ireland) immunochemical system. The total cholesterol, HDL cholesterol, LDL cholesterol, and triglyceride levels were determined using Cormay enzyme kits (Cormay, Lublin, Poland). The urinary albumin excretion level was quantified through an immunoturbidometric assay utilizing the Tina-quant (Boehringer Mannheim GmbH, Mannheim, Germany) system. Serum creatinine levels were assessed with the CREA system (Boehringer Mannheim GmbH).

The plasma concentrations of ICAM-1, sVCAM-1, and sP-selectin were determined using enzyme-linked immunosorbent assay (ELISA) kits (R&D Systems Minneapolis, MN, USA). In this study, the intraassay coefficients of variation were 2.8% for sP-selectin, 4.3% for ICAM-1, and 3.3% for sVCAM-1. The lower detection limits of the assays were 1.5 ng/mL for sP-selectin, 5.1 ng/mL for ICAM-1, and 33.8 ng/mL for sVCAM-1. The absorbance levels of ICAM-1, P-selectin, and sVCAM-1 were read at 450 nm on a CHROMATE 4300 automatic plate reader (Awareness Technology, Inc. USA, Minneapolis, MN, USA). The reference curve was prepared based on the manufacturer’s recommendations.

### 2.3. Capillaroscopy

Skin microcirculation was assessed by capillaroscopy using specialized software [8]. Two weeks prior to the onset of the study, participants were instructed to refrain from any activity that could damage the fingernail.

During the study, patients remained seated with a comfortably supported arm and hand under the capillaroscope. Body temperature was monitored with a non-contact thermometer (Novama-NT 19) and was within normal range in all patients studied. Images were taken with a digital camera (5MPx; OPTA-TECH, Warsaw, Poland) connected to a capillaroscope (OPTATECH, CS-CREATIVE SOLUTIONS Group, Warsaw, Poland) and archived on a disc. Image analysis was used to determine the ratio of the visible capillary area to the total analyzed image area (coverage) [8,25].

To assess cutaneous microcirculation reactivity, the PORH test was carried out following a period of rest lasting 20 min. A blood pressure cuff was placed around the patient’s non-dominant arm and inflated to 50 mm Hg above systemic systolic pressure to completely occlude the blood flow for 4 min [26]. Capillary images were taken immediately before (Coverage_BASE_) and after PORH (Coverage_PORH_). The reactivity of the micro-circulation was assessed as the change in the coverage during the test. A positive value for the difference between Coverage_PORH_ and Coverage_BASE_ (denoted as ∆coverage) was taken to indicate a reactive microcirculation, while a negative value was taken to indicate a non-reactive microcirculation.

### 2.4. Pulsatility Indices and Ankle–Brachial Index Examination

The pulsatility testing of the carotid, brachial, and lower extremity arteries was conducted with the VasoGuard 5000 device (Nicoletes, Image Monitoring Inc., Mississauga, ON, Canada) following 10 min of adaptation in the supine position and a test duration of 30 min. The methodology of the study with Vasoguard was previously elucidated in great detail [27].

The cuffs were placed on the lower extremities at four distinct anatomical locations: the upper thigh, above the knee, below the knee, and above the ankle. The cuffs were also applied to both arms. The assessments of pulsatility indices were simultaneously performed on the upper and lower limbs on both sides.

The device’s software allows for the determination of the pulsatility index (PI) for all arteries tested. Measurements on the common carotid artery were taken three times on each side, after which the mean pulsatility was calculated according to the Gosling formula [27,28].

The ankle- brachial index (ABI) was determined automatically using the VasoGuard device (Nicoletes, Image Monitoring Inc., Mississauga, ON, Canada) for both the upper and lower extremities simultaneously.

### 2.5. Optical Coherence Tomography

The early retinal changes were assessed using the optical coherence tomography (OCT) technique with (Topcon, Tokyo, Japan) instrumentation [29]. The mean cube thickness (TAC) and central subfield thickness (CST) were quantified using subfields according to the Early Treatment Diabetic Retinopathy Study (ETDRS) protocol. TAC in OCT scans represents the mean thickness of the retina, as measured over a cube-shaped area within a three-dimensional scan. The volume cube in OCT represents the total volume of the retina within a specific three-dimensional area imaged by the scan. This method allows the calculation of the volume of retinal tissue present within the scanned area. This is achieved by multiplying the thickness of each point by the area covered by the scan. In OCT, the term “central subfield thickness” (CST) refers to the average thickness of the retina present within the central macular zone of a circle with a diameter of 1 mm, situated in the center of the fovea. TAC and CST values were obtained by averaging the data for the left and right eyes [30].

### 2.6. Statistical Analysis

All data obtained underwent statistical analysis using STATISTICA version 13.1, a statistical software (StatSoft Inc., Tulsa, OK, USA). The software was licensed to CSM GUMed under the license number JPZP5077539317AR-H.

The distribution of variables was assessed using the Shapiro–Wilk test. As the variables did not follow a normal distribution, a non-parametric approach was adopted, employing the Mann–Whitney U test. For variables demonstrating a normal distribution, indicated as mean value (SD—standard deviation), parametric tests, specifically the Student’s *t*-test, were conducted for comparison. The interrelationships among variables were assessed utilizing Spearman’s rank correlation and a Chi-squared test with Yates correction when applicable. The Chi-squared test was employed to compare gender distributions, Tanner scale distributions, and the incidence of hypoglycemic episodes and autoimmune diseases. The influence of covariates was studied using the ANCOVA procedure. The study population was selected following the established criteria, and all variables were analyzed for the entire group, without excluding any outliers. A significance threshold of *p* < 0.05 was deemed to indicate statistical significance.

## 3. Results

### 3.1. Ages Versus Micro- and Macrocirculation Parameters

A positive correlation was observed between the age of the patients and the pulsatility indices assessed on the muscular arteries, including brachial (r = 0.39, *p* = 0.003), thigh (r = 0.48, *p* = 0.004), above (r = 0.44, *p* = 0.001) and below the knee (r = 0.53, *p* < 0.001), and at ankle level (r = 0.56, *p* < 0.001). A correlation was identified between the duration of diabetes and the pulsatility of arteries below the knee (*p* = 0.35, r = 0.009). However, no such correlation existed between the age of onset and any of the evaluated parameters describing microcirculation and macrocirculatory function.

### 3.2. Microcirculation Versus Macrocirculation

**Figure 1:**
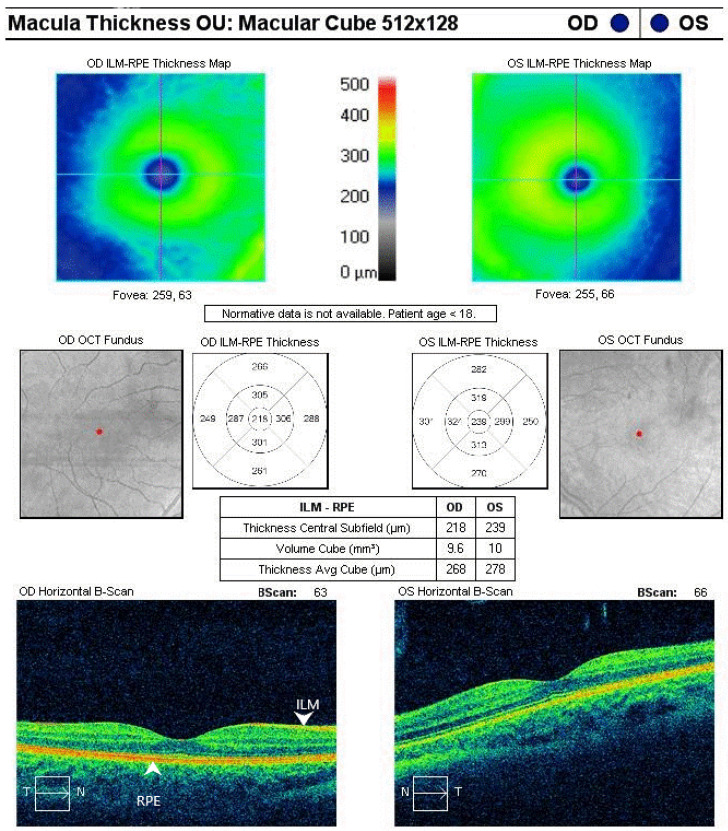
The result of the optical coherence tomography (OCT) examination of a subject with T1DM who participated in the present study. Macula thickness OU—the abbreviation *OU* is derived from the Latin term oculus uterque, which translates to “both eyes”. Retinal thickness is measured as the distance between the internal limiting membrane (ILM) and the retinal pigment epithelium (RPE)- white arrows. OD—right eye; OS—left eye.

We found a positive correlation between CST and both carotid_PI (r = 0.29, *p* = 0.03) and brachial_PI (r = 0.27, r = 0.04) (Table 1 and Figure 1).

### 3.3. Metabolic Parameters Versus Micro- and Macrocirculation

The analysis of the relationship between the parameters characterizing micro- and macrocirculation and the indices of metabolic compensation in the patient cohort revealed a significant negative correlation between CCA_PI and the levels of total cholesterol (r = −0.44, *p* = 0.001) and LDL cholesterol (r = −0.38, *p* = 0.004), in addition to the HDL fractions (r = −0.28, *p* = 0.04). Additionally, there was a significant negative correlation between muscular artery pulsatility, assessed at the arm and thigh, and both total cholesterol (r= −0.35, *p* = 0.01; r = −0.27, *p* = 0.03, respectively) and LDL fraction (r = −0.32, *p* = 0.02; r = −0.29, *p* = 0.0498, respectively). Conversely, there was a significant positive correlation between ABI and HDL cholesterol levels (r = 0.28, *p* = 0.04). The analysis yielded no statistically significant correlations between the examined parameters describing the arteries and the HbA_1c_ level. No correlation with the HbA_1c_ or lipid levels was found for any cutaneous microcirculation parameters analyzed.

### 3.4. Metabolic Parameters Versus Adhesion Molecules

The correlation between the levels of selected adhesion molecules and the parameters of metabolic compensation revealed a significant negative correlation between sVCAM-1 levels and total cholesterol (r = −0.37, *p* = 0.006), as well as LDL fraction cholesterol (r = −0.35, *p* = 0.009).

### 3.5. Adhesion Molecules Versus Micro- and Macrocirculation Parameters

The correlation between indices assessing cutaneous microcirculation, retinal micro-circulation, and indices of large vessel function and specific adhesion molecules was analyzed. The results demonstrated a significant and strong inverse correlation between the change in capillary coverage in the PORH test (∆coverage) and sP-selectin levels (r = −0.42, *p* = 0.001). Furthermore, a noteworthy positive correlation was observed between the CCA_PI value and the sVCAM-1 concentration (r = 0.29, *p* = 0.03). Nevertheless, no other examined parameter that concerned micro- or macrovascular function was found to correlate significantly with the selected adhesion molecule levels (Table 2).

### 3.6. Subdivision Based on Skin Microcirculation Reactivity (∆Coverage)

The study population included 39 young patients with type 1 diabetes mellitus who were non-reactive (group NR, ∆coverage ≤ 0) in the capillaroscopy and post-occlusive hyperemia tests. The remaining 16 patients with T1DM were reactive (group R, ∆coverage > 0) in these tests. To ensure comparability between groups, patients were matched for gender, age, and duration of diabetes. The age of onset in the cohort displaying non-reactive microcirculation was found to be 8.4 ± 3.5 years, whereas in the comparison group, it was 6.4 ± 3.1 years (Table 3). There was a marginal difference in the age of disease onset between the two groups: R and NR (*p* = 0.048).

The study found no significant difference in the distribution of puberty stage (according to the Tanner scale) between the R and NR groups (*p* = 0.16). The diabetic groups did not differ significantly concerning patient BMI, current HbA_1c_, treatment with a pump, duration of insulin pump use or insulin dose, or the number of mild and severe hypoglycemia episodes (Table 3). Additionally, no notable discrepancies were observed between the R and NR groups concerning heart rate and blood pressure.

The comparison of the R and NR groups revealed no noteworthy differences in the lipid profile, thyroid hormones, creatinine level, CRP, or albuminuria. Moreover, no notable discrepancies were identified in the concentration of sVCAM-1 and ICAM-1 between the two groups. The only significant differences were observed in selectin levels. The level was found to be significantly higher in the group with a non-reactive microcirculation (see Table 4).

The analysis of differences between subgroups separated by cutaneous microcirculatory reactivity showed no significant differences in parameters describing retina, carotid, and peripheral arterial pulsatility and ABI values (Table 5).

After adjustment for age at disease onset, the difference in P-selectin levels between the two groups remained statistically significant, albeit with a higher mean level of P-selectin observed in the group without cutaneous microcirculation reactivity.

## 4. Discussion

Pathophysiological processes in type 1 diabetes accelerate the development of atherosclerosis. As the early prevention of primary disease is beneficial for patients, researchers are looking for simple indicators to detect endothelial dysfunction and predict the development of vascular complications, even in pediatric patients and those who do not present poor metabolic compensation, as assessed by HbA_1c_ or lipid profile.

E-selectin and P-selectin are adhesion molecules that are expressed on the surface of inner-membrane endothelial cells and platelets. These molecules facilitate the rolling of leukocytes along the vessel wall until Intercellular Adhesion Molecule-1 and Vascular Cell Adhesion Molecule-1 induce robust adhesion and subsequent passage of leukocytes through the endothelial layer [17]. P-selectin has been identified as a potential biomarker of arterial and venous thrombosis [31,32], particularly that associated with cancer [33,34]. The role of P-selectin in the pathogenesis of atherosclerosis has been indicated in several studies [35,36,37]. In a prospective study of subjectively healthy women, Ridker et al. observed that those who developed cardiovascular events 3.5 years earlier had significantly elevated P-selectin levels [38]. In another study of atherosclerosis in children with type 1 diabetes, Simeunovic et al. observed that early inflammation present five years after diagnosis persists for ten years of disease duration, with moderate changes in most inflammatory markers over time, including P-selectin. The study involved children with an average age of 13.7, a disease duration of 5.6 years, and an average HbA1c of 8.4% [39].

The present study examined the relationship between levels of selected adhesion molecules and cutaneous microcirculatory disorders in uncomplicated young patients with type 1 diabetes. The cutaneous microcirculatory status of patients was evaluated using quantitative capillaroscopy and the post-occlusive reactive hyperemia (PORH) test. The patients exhibiting reactive microcirculation were defined as those with post-test capillary coverage exceeding the baseline value. Our findings indicated that in young patients with type 1 diabetes, a reduction in cutaneous microcirculatory reactivity was linked to markedly elevated levels of P-selectin. The difference between the two groups did not change when the data were adjusted for the age of onset.

A study by Kuryliszyn et al. [7] demonstrated a correlation between serum sE-selectin levels in adult patients with type 1 diabetes and the presence of abnormalities in capillaroscopy images. Higher sE-selectin levels in adult patients with type 1 diabetes have also been shown by other researchers [40]. In the EURODIAB Prospective Complication Study in patients with type 1 diabetes, sE-selectin, and sVCAM-1 serum levels were positively correlated with retinopathy, albuminuria, and cardiovascular disease [41]. A positive correlation was found between elevated sVCAM-1 level and the later onset of T1D, but no such correlation was observed concerning long-term diabetes complications [42]. The research conducted by Głowińska et al. has demonstrated that in children with obesity, hypertension, and diabetes, elevated levels of sICAM-1, sVCAM-1, and E-selectin are indicative of endothelial activation, a predictor of the early stages of atherosclerosis [43]. Similar results were obtained in a Norwegian study of 314 children with type 1 diabetes, with a mean age of 13.7 years, disease duration of 5.5 years, and HbA1c of 8.4%. This study showed that the levels of ICAM-1 and VCAM-1 were significantly higher than in the control group [11]. In another study, in a group of young patients with type 1 diabetes aged 7 to 20 years, ICAM-1 and VCAM-1 levels were not associated with CIMTs (carotid intima-media thickness), but CIMTs and ICAM-1 levels were significantly higher in patients with diabetes. The authors suggest that CIMT and ICAM-1 may be used to detect early atherosclerosis in children and adolescents with T1DM [44]. However, Glowinska-Olszewska et al. reported that adolescents with diabetic retinopathy had thicker IMT, but adhesion molecule levels were comparable to those in patients without retinopathy [45].

Systemic circulatory changes may affect microcirculatory function. Consequently, the impact of altered mechanical properties of the arterial wall, the character of flow, and blood viscosity is a principal area of research for scientists in this field. The commonly used pulsatility index is a readily available, simple, and non-invasive test suitable for use in pediatric populations. Several studies have demonstrated a correlation between arterial pulsatility and the development of atherosclerosis. Atherosclerosis is a process that starts at an early age, yet there is a lack of research on children, particularly those with type 1 diabetes. Consequently, the pulsatility index has been established as a valuable predictor of cardiovascular risk and the formation of atherosclerotic plaques. Jotoku et al. [46] have also identified a significant relationship between the pulsatility index of the common carotid artery and several cardiovascular risk factors, including homocysteine and monocyte levels. These are associated with the progression of atherosclerotic plaque formation in the coronary arteries and myocardial infarction. Significantly elevated renal artery resistance and pulsatility indices were observed in the pediatric population with type 1 diabetes, particularly in those with concomitant diagnoses of insulin resistance syndrome, hypertension, and obesity [47]. In a group of adult patients with T1DM, Kozera et al. showed the simultaneous presence of cutaneous microangiopathy and cerebral microangiopathy, as indicated by an increased PI of the middle cerebral artery. However, they found no significant correlation between the function of the two microvascular areas [12].

The present study demonstrated that the pulsatility indices of all studied arteries, both elastic and muscular, did not differ between subgroups, defined on the basis of cutaneous microcirculation reactivity. Nevertheless, a noteworthy positive correlation was found between the level of sVCAM-1 and the pulsatility indices of the common carotid and brachial arteries.

A few studies have indicated that the relationship between endothelial dysfunction and vascular stiffening may not be a simple cause-and-effect phenomenon. There may be an inverse relationship with vascular stiffening contributing to endothelial dysfunction. It can thus be postulated that a vicious cycle may exist whereby stiffening leads to endothelial dysfunction, which in turn worsens stiffening [48]. These and other studies suggest that the compliance of the vascular wall affects endothelial mechanical transduction and that wall stiffness may promote a decrease in NOS activity, leading to vascular stiffening [49].

The findings of our study suggest that certain younger patients demonstrated notable endothelial impairment and alterations in the cutaneous microcirculation. However, these changes have not yet led to a significant increase in pulsatility. There were no significant differences in hyperglycemia-induced changes in the artery wall in the studied vessels. It should be noted that comparable pulsatility occurred between groups with different skin microcirculation reactivity. The identification of P-selectin as a predominant factor indicates that younger diabetic patients exhibited a more pronounced endothelial activation process, which was associated with impaired cutaneous vascular reactivity.

Retinal OCT findings were similar in our studied groups of T1DM patients with differing cutaneous microvascular reactivity, suggesting that cutaneous angiopathy may precede the onset of diabetic retinopathy. Therefore, monitoring cutaneous microcirculation reactivity as a part of routine outpatient care appears to offer clinical value.

A limitation of the current study is the relatively small sample size. The inability to refer to patients who have experienced vascular complications and who are of a similar age and duration of diabetes onset also presents a challenge in formulating conclusions. The study group was not large enough to allow such comparisons.

## 5. Conclusions

Elevated levels of P-selectin may be a potential immunological marker of cutaneous abnormalities, which are an early indicator of endothelial dysfunction in young patients with type 1 diabetes without classical complications.

## Figures and Tables

**Table 1:** The correlation between circulation indices in the entire cohort of patients with diabetes mellitus.

Circulation Indices	CST		TAC		Coverage_BASE_		Coverage_PORH_		∆Coverage	
	r	*p*	r	*p*	r	*p*	r	*p*	r	*p*
CST	-	-	-	-	0.00	0.99	−0.03	0.84	−0.05	0.71
TAC	-	-	-	-	0.12	0.39	0.00	0.98	−0.18	0.19
ABI	−0.13	0.33	0.05	0.74	0.20	0.15	0.18	0.19	0.00	0.99
CCA_PI	0.29	0.03	−0.12	0.37	0.06	0.64	0.08	0.54	0.06	0.67
brachial_PI	0.27	0.04	0.05	0.72	0.03	0.85	0.04	0.78	0.02	0.89
thigh_PI	0.08	0.56	−0.17	0.22	−0.07	0.60	−0.03	0.85	0.06	0.69
above_knee_PI	0.18	0.19	−0.02	0.86	−0.09	0.50	−0.03	0.80	0.04	0.78
below_knee_PI	0.25	0.07	−0.06	0.66	−0.06	0.64	0.01	0.92	0.06	0.65
ankle_PI	0.18	0.19	0.01	0.94	−0.01	0.96	−0.01	0.93	0.004	0.98

Abbreviations: r—Correlation coefficient; CST—central subfield thickness; TAC—Thickness Average Cube; Coverage_BASE_—the surface area covered by capillaries before the PORH test; Coverage_PORH_—the surface area covered by capillaries after the PORH test; ∆Coverage—the difference in surface area covered by capillaries before and after the PORH test; ABI—ankle–brachial index; CCA_PI—pulsatility index for carotid artery; brachial_PI—pulsatility index for brachial artery; thigh_PI—pulsatility index for femoral artery; above_knee_PI—pulsatility index for muscular artery above knee; below_knee_PI—pulsatility index for muscular artery below knee; ankle_PI—pulsatility index for muscular artery at the ankle level. The interrelationships among variables were assessed utilizing Spearman’s rank correlation. The value of *p* < 0.05 was regarded as statistically significant.

**Table 2:** Correlations between sP-selectin, sVCAM-1, ICAM-1, and circulation indices in the entire cohort of patients with diabetes mellitus.

Adhesion Molecules	sP-Selectin		sVCAM-1		ICAM-1	
Circulation Indices	r	*p*	r	*p*	r	*p*
CST	0.10	0.46	0.23	0.09	0.01	0.95
TAC	−0.03	0.84	−0.11	0.41	−0.23	0.10
Coverage_BASE_	0.18	0.18	0.02	0.90	0.13	0.35
Coverage_PORH_	−0.15	0.28	−0.12	0.38	0.05	0.71
∆Coverage	−0.42	0.001	−0.11	0.41	−0.06	0.64
ABI	−0.03	0.82	−0.05	0.72	0.19	0.17
CCA_PI	0.10	0.47	0.29	0.03	0.12	0.37
brachial_PI	0.16	0.25	0.29	0.03	0.24	0.08
thigh_PI	−0.11	0.42	0.10	0.47	0.12	0.38
above_knee_PI	−0.15	0.27	0.00	0.98	0.06	0.66
below_knee_PI	−0.10	0.45	−0.04	0.75	0.15	0.27
ankle_PI	−0.08	0.58	−0.10	0.45	0.15	0.27

Abbreviations: r—Correlation coefficient; sVCAM-1—Soluble Vascular Cell Adhesion Molecule-1; ICAM-1—Intercellular Adhesion Molecule-1; sP-Selectin—Soluble Platelet Selectin; CST—central subfield thickness; TAC—Thickness Average Cube; Coverage_BASE_—the surface area covered by capillaries before the PORH test; Coverage_PORH_—the surface area covered by capillaries after the PORH test; ∆Coverage—the difference in surface area covered by capillaries before and after the PORH test; CCA_PI—pulsatility index for carotid artery; brachial_PI—pulsatility index for brachial artery; thigh_PI—pulsatility index for femoral artery; above_knee_PI—pulsatility index for muscular artery above knee; below_knee_PI—pulsatility index for muscular artery below knee; ankle_PI—pulsatility index for muscular artery at the ankle level; ABI—ankle–brachial index. The interrelationships among variables were assessed utilizing Spearman’s rank correlation. The value of *p* < 0.05 was regarded as statistically significant.

**Table 3:** Comparison of characteristics of diabetic patients divided according to cutaneous reactivity presence.

Characteristics	T1DM Subgroups According to Cutaneous Reactivity		
	Non–Reactive (NR) n = 39	Reactive (R) n = 16	*p* for Between-Group Comparison
Males n (%)	16 (41)	10 (62.5)	0.25
Age [years]	15.5 (8.4–17.9) 15.3 ± 2.3	15.2 (11.1–18) 15.3 ± 2.6	0.82
Onset of diabetes [age]	9.1 (2.1–13.6) 8.4 ± 3.5	6.3 (1.2–12.7) 6.4 ± 3.1	0.048
T1DM duration [years]	6.9 (1.2–14.5) 6.9 ± 3.5	10.1 (1.7–14.6) 8.8 ± 3.9	0.08
BMI [kg/m^2^]	20.5 (14.5–29.7)	20.8 (15–26)	0.99
Insulin dose [units/24 h]	45 (21–75)	46 (20–100)	0.65
Time of pump treatment as a ratio to T1DM duration [%]	60 (0–100)	50 (0–100)	0.88
HbA_1c_ [%]	7.8 (5.9–11.6)	9 (6.2–13.4)	0.09
Episodes of mild hypoglycemia [N/last month]	10 (0–30)	10 (3–20)	0.93
Episodes of severe hypoglycemia [N/last year]	0 (0–2)	0 (0–1)	0.93
Autoimmune disease, n [%]	10 (25.6)	6 (37.5)	0.58
SBP [mmHg]	107 (89–124)	107 (94–126)	0.83
DBP [mmHg]	60 (49–76)	59 (49–71)	0.58
HR [beats/min.]	79 (65–114)	82 (57–99)	0.93

Abbreviations: T1DM—type 1 diabetes mellitus; HbA_1C_—glycated hemoglobin; BMI—body mass index; Autoimmune disease—autoimmune thyroiditis and/or celiac disease; SBP—systolic blood pressure; DBP—diastolic blood pressure; HR—heart rate. The distribution of variables was assessed using the Shapiro–Wilk test (a normal distribution) and the Mann–Whitney U test (not normally distributed). Values are presented as median (minimum; maximum) and mean ± SD. The value of *p* < 0.05 was regarded as statistically significant.

**Table 4:** Comparison of laboratory results of diabetic patients divided according to cutaneous reactivity presence.

Characteristics	T1DM Subgroups According to Cutaneous Reactivity		
	Non-Reactive (NR) n = 39	Reactive (R) n =16	*p* for Between-Group Comparison
Total cholesterol [mg/dL]	178 (125–288)	184 (135–269)	0.64
Cholesterol LDL [mg/dL]	106 (61–188)	110 (61–180)	0.58
Cholesterol HDL [mg/dL]	57 (33–90)	54 (35–72)	0.64
Triglycerides [mg/dL]	72 (34–294)	99 (38–269)	0.16
TSH [mIU/L]	1.7 (0.6–5.1)	2.2 (0.6–4)	0.47
fT4 [pmol/L]	12.5 (9–15)	12.5 (10.1–15)	0.85
Serum creatinine [mg/dL]	0.7 (0.5–1)	0.7 (0.5–0.9)	0.68
Albuminuria [mg/dl]	6 (2.5–24.4)	10.2 (2.5–28)	0.06
C–reactive protein [mg/L]	0.6 (0.1–4.9)	0.6 (0.1–4.8)	0.79
sVCAM–1 [ng/mL]	336 (77–684)	283 (109–533)	0.17
ICAM–1 [ng/mL]	244 (78–490)	193 (83–472)	0.26
sP-Selectin [ng/mL]	302 (109–732)	204 (98–456)	0.007

Abbreviations: T1DM—type 1 diabetes mellitus; LDL—low-density lipoproteins; HDL—high-density lipoproteins; TSH—thyroid-stimulating hormone; fT4—free thyroxine; sVCAM-1—Soluble Vascular Cell Adhesion Molecule-1; ICAM-1—Intercellular Adhesion Molecule-1; sP-Selectin—Soluble Platelet Selectin. The distribution of variables was assessed using the Mann–Whitney U test (not normally distributed). Values are presented as median (minimum; maximum). The value of *p* < 0.05 was regarded as statistically significant.

**Table 5:** Comparison of pulsatility indices, ABI, and optical coherence tomography (OCT) indices of diabetic patients divided according to cutaneous reactivity presence.

Characteristics	T1DM Subgroups According to Cutaneous Reactivity		
	Non-Reactive (NR) n = 39	Reactive (R) n = 16	p for Between-Group Comparison
Cutaneous microcirculation			
Coverage_BASE_ [%]	16.9 (12.7–24.8)	15.9 (13.4–20.1)	0.21
Coverage_PORH_ [%]	15.3 (10.3–24.4)	18.3 (14–24.3)	0.002
∆Coverage [%] *	–1.3 (–7.3–0)	1.4 (0.2–4.9)	<0.001
Pulsatility indices and ABI			
CCA_PI	1.88 (1.37–3)	1.81 (1.44–2.64)	0.41
brachial_PI	2.43 (1.81–3.74)	2.28 (1.97–3.04)	0.51
thigh_PI	2.29 (1.92–3.08)	2.20 (2.02–2.93)	0.31
above_knee_PI	2.28 (1.9–3.21)	2.19 (2.03–3.48)	0.61
below_knee_PI	2.51 (2.07–3.62)	2.57 (2.17–3.26)	0.95
ankle_PI	2.49 (0–3.45)	2.40 (1.96–3.06)	0.46
ABI	1.1 (0.8–1.3)	1.1 (1–1.3)	0.51
OCT indices			
CST	250 (213–309)	250 (208–277)	0.73
Volume cube	10.2 (9.6–11.1)	10.2 (9.6–10.7)	0.30
TAC	285 (268–310)	285 (266–298)	0.32

Abbreviations: T1DM—type 1 diabetes mellitus; Coverage_BASE_—the surface area covered by capillaries before the PORH test; Coverage_PORH_—the surface area covered by capillaries after the PORH test; ∆Coverage—the difference in surface area covered by capillaries before and after the PORH test; CCA_PI—pulsatility index for carotid artery; brachial_PI—pulsatility index for brachial artery; thigh_PI—pulsatility index for femoral artery; above_knee_PI—pulsatility index for muscular artery above knee; below_knee_PI—pulsatility index for muscular artery below knee; ankle_PI—pulsatility index for muscular artery at the ankle level; ABI—ankle–brachial index; CST—central subfield thickness; TAC—Thickness Average Cube; OCT—optical coherence tomography. The distribution of variables was assessed using the Mann–Whitney U test. Values are presented as median (minimum; maximum). The value of *p* < 0.05 was regarded as statistically significant. *—criterion for splitting the group.

## Data Availability

The data presented in this study are available on request from the corresponding author.
